# Sensitizing Temozolomide-resistant Glioblastoma to Ferroptosis with High-dose L-ascorbic acid

**DOI:** 10.51894/001c.164000

**Published:** 2026-07-31

**Authors:** Visakuo Tsurho, Joey Norton, Leif Neitzel, Charles Williams, Charles C. Hong

**Affiliations:** 1 College of Osteopathic Medicine, Michigan State University

## 06

### Introduction

L-ascorbic acid (L-AA) is an emerging and promising therapeutic adjunct to chemotherapy. At high doses, L-AA exhibits pro-oxidative properties that selectively eliminate cancer cells and enhance the efficacy of traditional chemotherapies. Notably, high-dose L-AA improves immune cell function and mitigates chemotherapy toxicity, offering an attractive avenue to improve patients’ quality of life during cancer treatment.

Though some cancers are resistant to L-AA combination therapies, they may be susceptible to ferroptosis induction. Ferroptosis is an iron-mediated cell death pathway that eliminates most chemotherapy-resistant cancers. High-dose L-AA can sensitize cancer cells to ferroptosis inducers such as erastin. Unfortunately, traditional ferroptosis inducers are cytotoxic to healthy cells, making them undesirable for clinical application.

To overcome this challenge while leveraging the bifunctional benefits of L-AA in glioblastoma, I investigated the synergistic potential of L-AA with ogremorphin (OGM). Unlike other ferroptosis inducers, OGM induces ferroptosis by specifically inhibiting GPR68, a proton-sensing GPCR selectively upregulated in glioblastoma. I evaluated the efficacy of L-AA in sensitizing temozolomide-resistant U138 glioblastoma cells to OGM and other ferroptosis inducers.

### Objectives

Sensitize glioblastoma cells to ferroptosis inducers using L-AA and elucidate the underlying ferroptosis regulatory network.

### Methods

U138 glioblastoma cells were treated with varying doses of L-AA (AAA1561322, Thermo Scientific Chemicals) for 72 hours, and a dose-survival curve was determined using Cell-Titer Glo (G7571, Promega) and Hoechst (H3570, Invitrogen). Subsequently, U138 cells were treated with Ogremorphin (8345, Michigan State University), Erastin (5449, Tocris), Temozolomide (2706, Tocris), and 1R,3R-RSL3 (6687, Tocris) +/- L-AA, and cell viability and metabolism were assessed.

### Results

High-dose L-AA impairs energy production and viability of U138 cells. High cell density is protective against L-AA-promoted ferroptosis (*p* < 0.0001). L-AA sensitizes U138 cells to OGM and RSL3-mediated ferroptosis (*p* < 0.0001). Data was analyzed by one-way ANOVA followed by Tukey’s post-hoc test.

### Conclusion

L-ascorbic acid is independently cytotoxic to Temozolomide-resistant glioblastoma cells and sensitizes them to mechanistically distinct ferroptosis inducers. L-AA also potentiates the effects of ogremorphin, a specific inhibitor of GPR68, a proton-sensing GPCR strategically upregulated in glioblastomas. Combination L-AA and OGM therapy may be the first-in-class ferroptosis-inducing drugs against refractory glioblastomas.

